# Supplementary figures and images for: Resurgence of malaria in Uganda despite sustained indoor residual spraying and repeated long lasting insecticidal net distributions
Source: PLOS Glob Public Health. 2022 Sep 7;2(9):e0000676. doi: 10.1371/journal.pgph.0000676 (PMC10022262; doi:10.1371/journal.pgph.0000676)

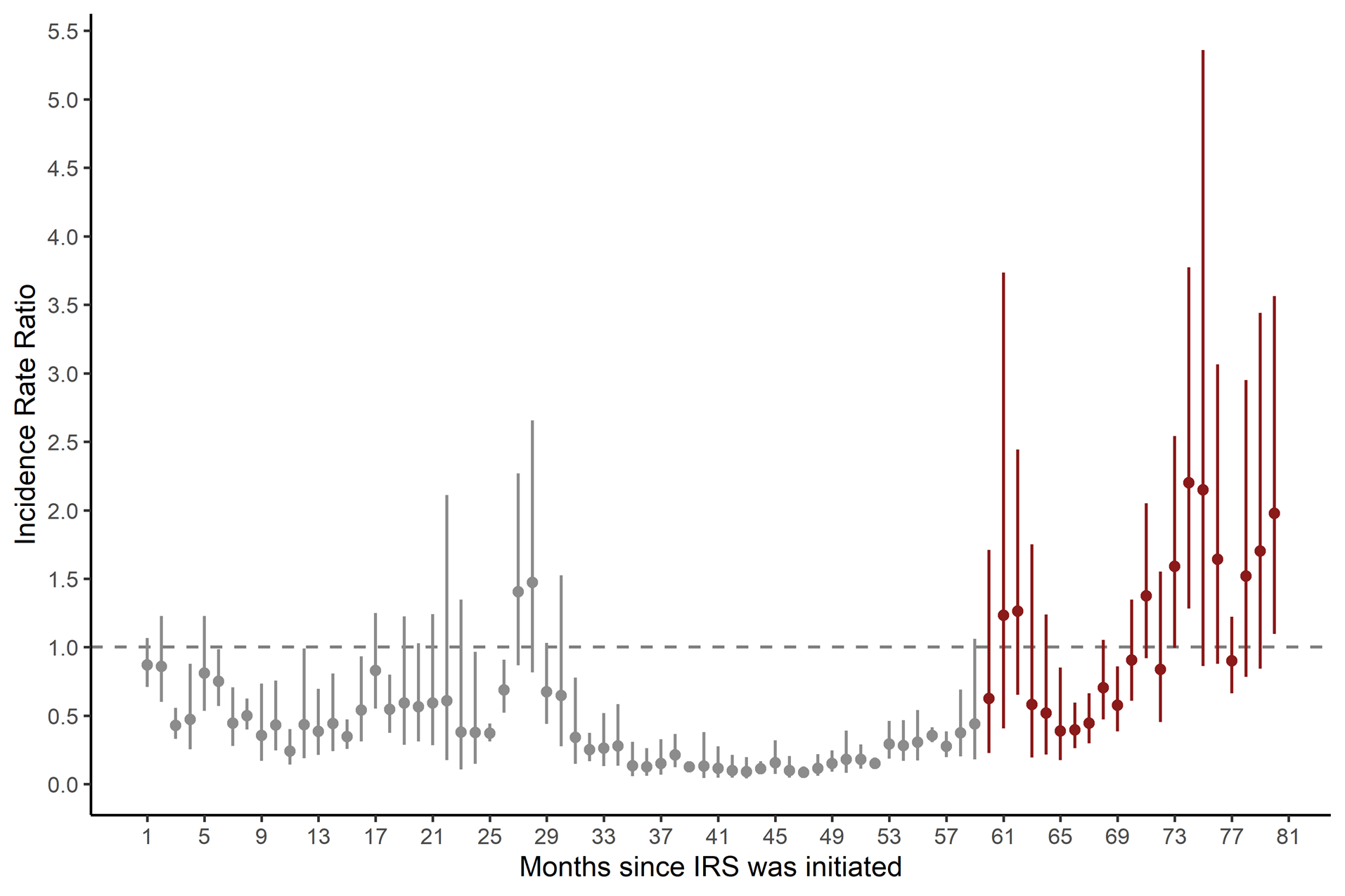

Supplement: S1 Fig — Vertical bars represent the 95% CI around adjusted IRR. Effect estimates in grey are published previously. (TIF) [file pgph.0000676.s001.tif]

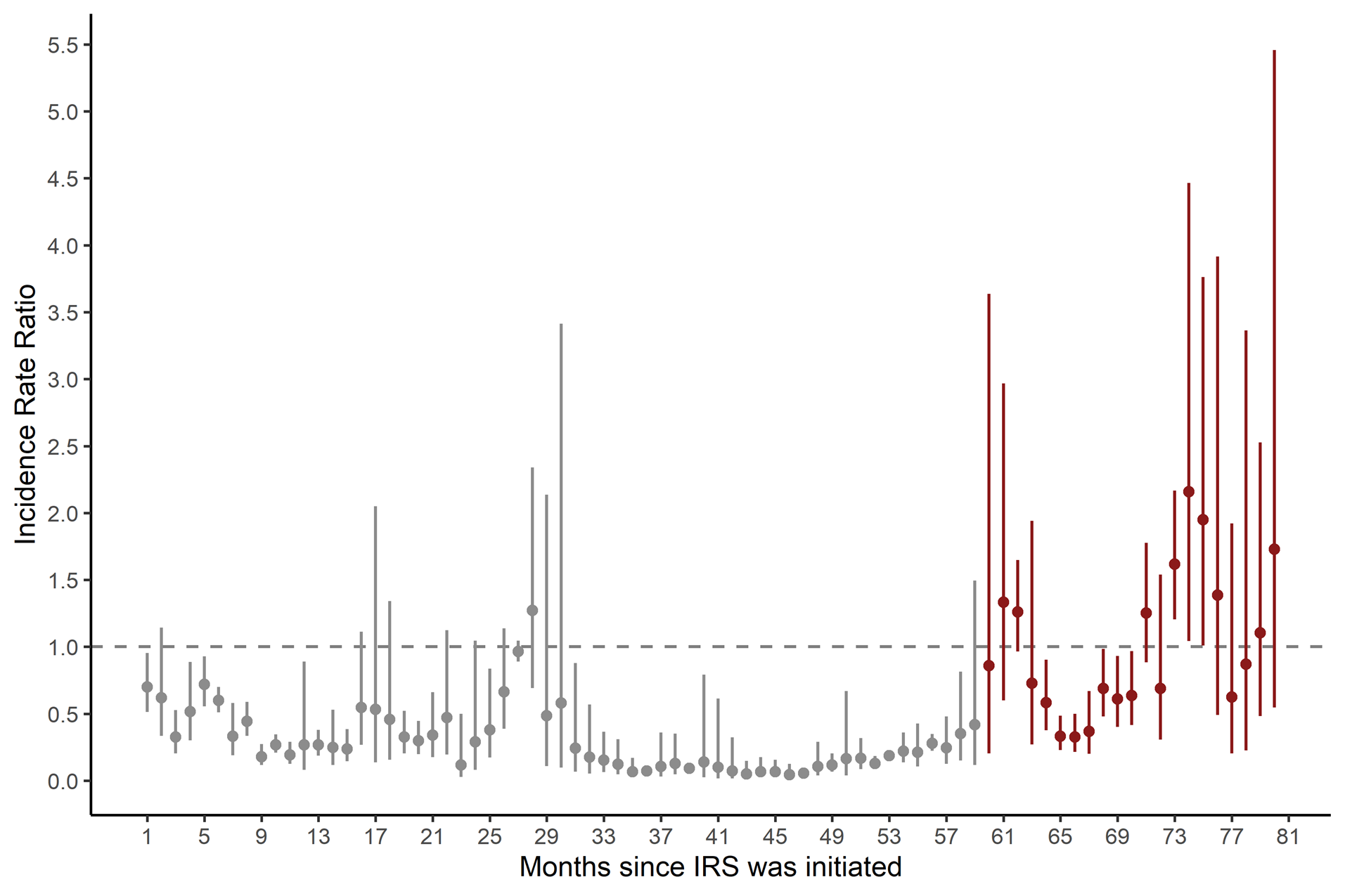

Supplement: S2 Fig — Vertical bars represent the 95% CI around adjusted IRR. Effect estimates in grey are published previously. (TIF) [file pgph.0000676.s002.tif]

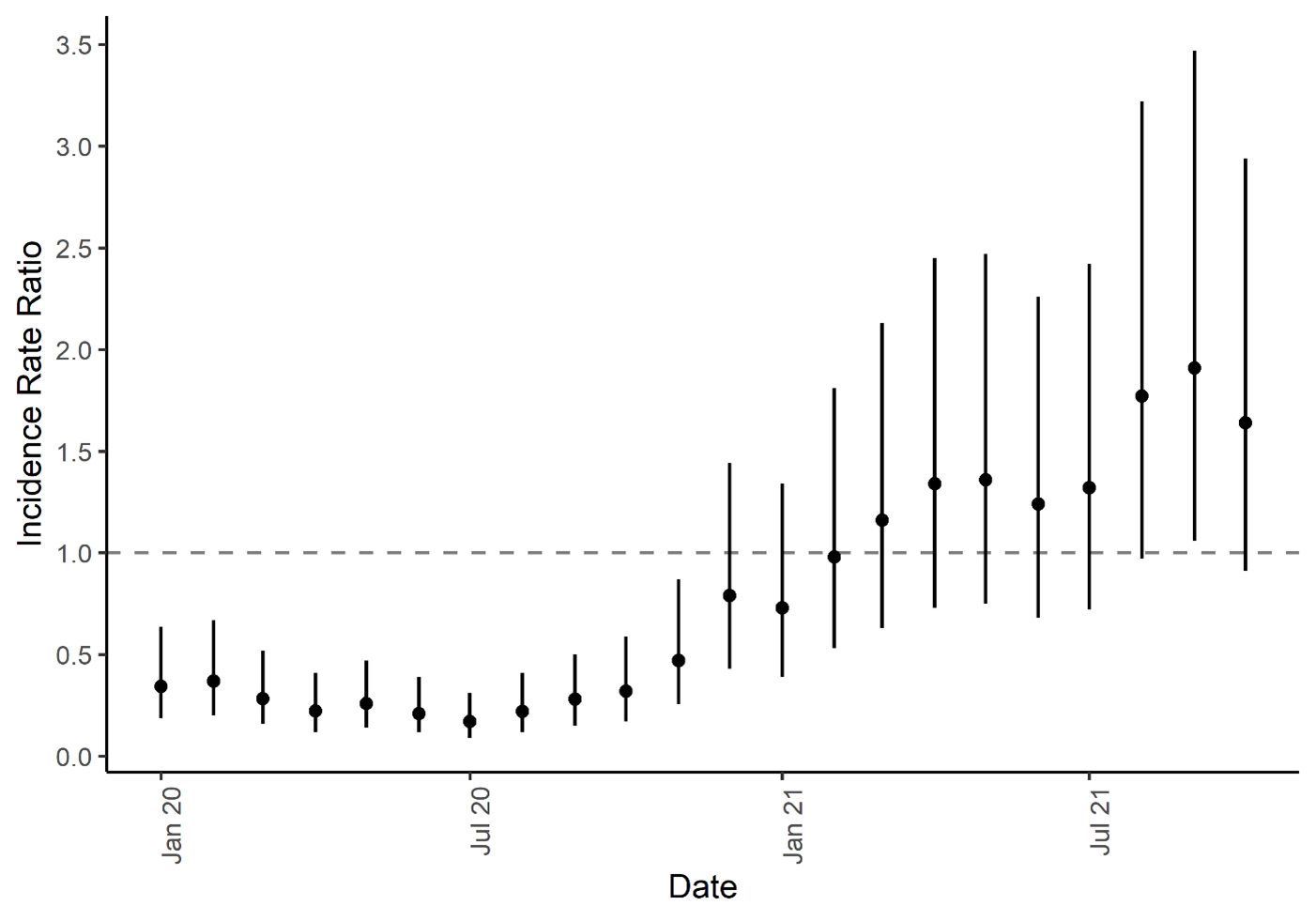

Supplement: S3 Fig — Vertical bars represent the 95% CI around adjusted IRR. (TIF) [file pgph.0000676.s003.tif]
