# Supplementary material for: Resurgence of malaria in Uganda despite sustained indoor residual spraying and repeated long lasting insecticidal net distributions
Source: PLOS Glob Public Health. 2022 Sep 7;2(9):e0000676. doi: 10.1371/journal.pgph.0000676 (PMC10022262; doi:10.1371/journal.pgph.0000676)
Supplement: Attachment — Submitted filename: IRS_Malaria_Resurgence_Response to reviewers_R1.docx [file pgph.0000676.s007.docx]

Journal Requirements:

1. Please amend your Financial Disclosure statement. If you did not receive any funding for this study, please simply state: “The authors received no specific funding for this work.”

**This has been amended.**

2. Please update your Competing Interests statement. If you have no competing interests to declare, please state: “The authors have declared that no competing interests exist.”

**This has been amended.**

3. We do not publish any copyright or trademark symbols that usually accompany proprietary names, eg (R), (C), or TM  (e.g. next to drug or reagent names). Please remove all instances of trademark/copyright symbols throughout the text, including Actellic 300CS®, SumiShield®, Fludora®, Permanet®, Royal Guard®, Royal Guard® on pages 4, 5, 6, 17, .

**These have been removed.**

4. All figures and supporting information files will be published under the Creative Commons Attribution License (creativecommons.org/licenses/by/4.0/). Authors retain ownership of the copyright for their article and are responsible for third-party content used in the article.

Figure 1: please (a) provide a direct link to the base layer of the map used and ensure this is also included in the figure legend; (b) provide a link to the terms of use / license information for the base layer. We cannot publish proprietary or copyrighted maps (e.g. Google Maps, Mapquest) and the terms of use for your map base layer must be compatible with our CC-BY 4.0 license.

If your map was obtained from a copyrighted source please amend the figure so that the base map used is from an openly available source. Alternatively, please provide explicit written permission from the copyright holder granting you the right to publish the material under our CC-BY 4.0 license.

Please note that the following CC BY licenses are compatible with PLOS license: CC BY 4.0, CC BY 2.0 and CC BY 3.0, meanwhile such licenses as CC BY-ND 3.0 and others are not compatible due to additional restrictions.

If you are unsure whether you can use a map or not, please do reach out and we will be able to help you. The following websites are good examples of where you can source open access or public domain maps: 
* U.S. Geological Survey (USGS) - All maps are in the public domain. (http://www.usgs.gov) 
* PlaniGlobe - All maps are published under a Creative Commons license so please cite “PlaniGlobe, http://www.planiglobe.com, CC BY 2.0” in the image credit after the caption. (http://www.planiglobe.com/?lang=enl) 
* Natural Earth - All maps are public domain. (http://www.naturalearthdata.com/about/terms-of-use/)

Please upload any written confirmation as an 'Other' file type. It must clarify that the copyright holder understands and agrees to the terms of the CC BY 4.0 license; general permission forms that do not specify permission to publish under the CC BY 4.0 will not be accepted. Note that uploading an email confirmation is acceptable.

**The background is a shapefile of the continent of Africa from GADM. Explicit agreement to the CC-BY 4.0 from GADM can be found here:** [**https://gadm.org/license.html**](https://gadm.org/license.html)

**This information has also been uploaded as an ‘Other’ file type.**

5. Please review your reference list to ensure that it is complete and correct. If you have cited papers that have been retracted, please include the rationale for doing so in the manuscript text, or remove these references and replace them with relevant current references. Any changes to the reference list should be mentioned in the rebuttal letter that accompanies your revised manuscript. If you need to cite a retracted article, indicate the article’s retracted status in the References list and also include a citation and full reference for the retraction notice.

**This has been done.**

Reviewer #1: Overall, I think this is an excellent manuscript that is very clearly written. It addresses two under-researched areas – the impact of IRS over multiple years and some evidence, albeit not strong, of the epidemiological impact of IRS with clothianidin-based products.

**Thank you very much, your comments below are much appreciated and strengthen the manuscript.**

I have only minor issues to report.
Minor issues:
1. Line 65-69: The first introduction paragraph is about the scale-up of LLINs and IRS, which doesn’t fit well with the main themes of the paper. I think the introduction would be stronger if it focused instead on the current evidence of what we know/don’t know about the evidence of IRS implementation. I would build on the mixed benefit statement and fill in other details re: we don’t know much about the duration (months)of impact from one campaign, impact of withdrawing the intervention, relative impact of IRS products, or the long-term impact of the interventions, beyond a few studies. This paper addresses the impact of 7 years of sustained IRS, addressing current gaps in the literature. Some of this content is included in the discussion and could be pulled into the introduction.

**We have added the following to the end of the first paragraph of the introduction section in response to this comment:**

***In addition, there are limited data on the impact of multiple rounds of IRS sustained over an extended period of time and the relative impact of switching to different formulations of IRS [5, 6]. This information is critical for maximizing the effectiveness of IRS, particularly as new products are deployed.***

2. Line 112: would be good practice to cite the WHO recommendation re: one ITN per 1 people.

**We have added this citation.**

3. Line 112: Would be helpful to name the specific pyrethroid chemical and brand for the conventional LLINs, as with the PBO and dual-AI LLINs.

**These details have been added.**

4. Line 131: Changing IRS insecticides every three years is not technically in line with WHO recommendations, although I understand why national programs take this approach due to the lack of options for new products. Per the WHO Guidelines Annex 9: “The pragmatic approach is to rotate insecticides annually. Changing insecticides more than once a year (which could be the case in areas where two spray rounds are conducted each year) is not recommended, mainly because of procurement and other logistical challenges. Changing less frequently than once a year is also not recommended, as using an insecticide for longer makes it more likely that resistance will evolve to a frequency that is too high for rotations then to be effective in reducing it.”

**Thank you for noting this. We have removed the phrase “in line with WHO recommendations” from this sentence and it now reads:**

***Changes of IRS insecticides were made in accordance with the Ugandan Ministry of Health’s insecticide resistance management plan which requires changing IRS formulations every three years to preempt the development of resistance [8, 9].***

5. Line 153: Could you explain why Study objective 2, with the non-IRS comparison sites only went back 2 years, creating a non-randomized post-only study design? Even though there is no randomization, I think it would be stronger if it looked at total trends in both IRS and non-IRS areas since the baseline period, but I understand if this is not possible because the data was not available.

**This is a great point that we will clarify in the manuscript. Indeed, only 1 of the 10 ‘non-IRS’ comparator sites have data going back to baseline. We therefore opted to just include the time period when the resurgence was observed. We have added the following to the manuscript to clarify:**

***We opted to compare malaria burden in IRS versus non-IRS sites only during years 6 and 7 of IRS implementation given the availability of data in non-IRS sites.***

6. Line 162: Would be nice to add a sentence about why the exposure variable was lumped into years 1-3, years 4-5, then separating out years 6 and year 7.

**We have added the following explanation to this paragraph:**

***Years 6 and 7 were presented separately as they represent data unpublished in our previous analysis (February 2020 through October 2021); we therefore believed temporal granularity was warranted.***

7. Line 276: The text says: “From December 2020 onward, the IRR crossed 1, indicating that cases at IRS sites were higher than non-IRS sites, although we could not rule out a null or negative association in these months.” Reading Figure 5, isn’t it that the upper bound confidence interval for the IRR crossed 1 and the IRR point estimate doesn’t cross 1 until March 2021. It would be helpful to clarify this in the text.

**Thank you for noting this error. We have rectified this in the text:**

***From December 2020 onward, the 95% CI for the IRR crossed 1, and from March 2021 onward the point estimate for the IRR crossed 1, indicating that cases at IRS sites were higher than non-IRS sites, although we could not rule out a null or negative association in these months.***

8. Line 323: The reasons for the increase in malaria cases could be reorganized to create a stronger argument. A clearer argument could be restructured as:
a. Clothianidin may not be as effective as pirimiphos-methyl (note Dokolo and only ento studies so far) Could add some information about the clothianidin mechanism and why it may not be as effective – that is slower acting than pirimiphos-methyl.
b. Increasing pyrethroid resistance may have rendered the 2020-2021 UCC campaign less effective than the 2017 campaign, resulting in a loss of the “dual impact” of IRS and LLINs. (cite trends in pyrethroid resistance 2017 to 2020-2021).
The statement on the loss of the combined impact of a dual-intervention with pirimiphos-methyl and pyrethroid LLINs isn’t very strong. There is still a combined intervention with a clothianidin product and pyrethroid LLINs. The issue could be the that pyrethroid LLINs are less effective and no longer contributing to the combined impact. This is why I would recommend removing this point and focusing in on the other two points above. Then I would consider a separate paragraph to discuss the comparative impact of clothianidin + pyrethroid LLINs vs. next generation LLINs. There is also no data on this comparison and I think this an important area that this paper hints at as well. Could next generation LLINs be a more cost-effective vector control intervention when compared with clothianidin + pyrethroid LLINs? This is a good area for future research.

**Thank you for these excellent recommendations. We have reorganized the Discussion as per your comments. We are pasting below the 2 edited paragraphs in question, one describing the potential that clothianidin is less impactful in this setting and a new paragraph discussing IRS+LLINs:**

***There are several potential factors that may be driving the increase in malaria burden in districts receiving IRS. First, the timing of the increase corresponds to a shift in active ingredient from pirimiphos-methyl to clothianidin-based formulations (primarily Fludora Fusion, a combination of clothianidin and deltamethrin). One reason clothianidin may be less effective than pirimiphos-methyl in this setting is that is slower-acting; if a proportion of mosquitoes exposed to clothianidin are surviving long enough to bite or lay eggs, this may lead to lower effectiveness of clothianidin compared to the fast-acting organophsopate pirimiphos-methyl. Of note, the one site (Dokolo) that received a single round of IRS with clothianidin-based SumiShield 50WG in 2019 (before the shift to Fludora Fusion WP-SB across all sites in 2020) experienced an increase in burden immediately following the round of SumiShield 50WG. Increases were not documented at other sites until the initiation of Fludora Fusion WP-SB in 2020. This is added evidence for the change in active ingredient contributing to the observed resurgence of malaria. To date, studies on the effectiveness of clothianidin alone (SumiShield ) [20-24] and clothianidin with deltamethrin (Fludora Fusion WP-SB) [20, 25-27] are limited to susceptibility studies focused on entomological outcomes, both in lab and in experimental huts. These studies document that clothianidin-based products succeed at killing mosquitoes, but none have evaluated clinically relevant outcomes in “real world” settings.***

***Another second potential driver that could explain the observed resurgence is that IRS districts received conventional pyrethroid LLINs in the 2020-2021 UCC campaign. This could be problematic given that wide-spread pyrethroid resistance has been described in Uganda [17-19] and may have increased since the 2017 UCC. Conversely, the 10 sites in neighboring districts where increases in burden were not observed received “next generation” nets designed for areas with wide-spread pyrethroid resistance. Therefore, the dual impact of IRS and LLINs in these districts where pyrethroid resistance is high or increasing may have been lost between the two LLIN distributions. Importantly, there are no data on the impact of IRS with clothianidin in combination with LLINs (pyrethroid or next-generation). This is a key area for future research.***

9. Line 365: Rather than just stating that is unlikely that mosquito behavior changed you could cite the recent PMI VectorLink entomological reports from 2019 and 2020, and perhaps other years, which include data from Lira and Otuke.

**This is a good point. We have added the following and cited the recommended PMI VL reports:**

***This, however, would be unlikely to have occurred rapidly and simultaneously at all IRS sites at a pace that would explain the resurgence observed in this study and is not echoed in PMI Vectorlink entomological surveillance reports from 2019 and 2020, which indicate that IRS is not associated with increases in outdoor biting [33, 34].***

10. Line 372: I think these are great references, but a bit confusing to the argument. The near collapse of Anopheles gambiae and Anopheles funestus data is from 2016. It makes one curious as to what these trends look like in the 2020-2021 ‘resurgence’ era compared to previous years. Could this data be gleaned from existing published literature or PMI VectorLink entomological reports over time?

**Thank you for this recommendation. We have added the following and removed the phrase mentioning the 2016 data:**

***However, recent data demonstrate that Anopheles gambiae and Anopheles funestus remain the primary vectors in Uganda [28], including in IRS districts (including Otuke and Tororo) according to 2020 entomological surveillance data [34].***

11. Line 391: Entomological measures and residual efficacy data is available from at least some entomological sites within the study areas through PMI VectorLink entomological reports.

**Thank you for pointing us to this. We have added the following:**

***Despite these limitations, this analysis has important scientific and policy implications. First, additional research is needed on the driving factors contributing to the observed resurgence of malaria burden in IRS districts, including targeted entomology and residual efficacy. Data from PMI Vectorlink in 2020 in Tororo district suggest that Fludora Fusion effectiveness extends up to 8 months depending on the wall type, with lower effectiveness on mud walls [34]; however, more data, with both greater spatial and longitudinal variation, are needed.***

12. S1: Should explain in the notes how coverage is defined. Is it structures sprayed out of structures found, or population protected out of total population? There doesn’t seem to be a standard definition in the literature so it would be good to clarify.

**We have added a footnote to the table indicating that coverage is defined as the percent of structures sprayed out of structures found.**

13. The authors note that the comparison study objective 2, while focused on IRS, is actually IRS + pyrethroid LLINs vs. next generation LLINs. Do you have documentation on the LLIN coverage in these districts or the relative coverage in the pyrethroid LLIN vs. next generation LLIN districts? The text states that nationally coverage was high, but coverage can vary by area. It would be helpful to note in the manuscript either the coverage rates across the pyrethroid LLIN vs. next generation LLIN districts or that the data was not available at this level.

**This is a good point. District-level data indicates that all districts included in this analysis had equally high coverage. We have indicated that in the discussion:**

***In Uganda, the LLIN distribution campaign was delayed by 5 months but successfully distributed over 28 million nets, achieving over 90% coverage; in both IRS and non-IRS districts included in this analysis coverage was greater than 97%.***

14. Throughout the paper the terminology changes from “sites” to “health facilities” to “Malaria Reference Centers”. It might be nice to standardize this terminology if possible.

**We have standardized this term to “site” where appropriate.**

Reviewer #2: • Please clearly state, define, and use the primary outcome measures instead of using vague terms such as “malaria burden”, “burden of malaria” throughout the manuscript, importantly in the abstract.

• It’s essential to add a table or two clearly showing the crude and adjusted IRR for both objectives 1 and 2, including only the lab-confirmed cases. The summary statistics presented in Tables 1 and 2 could be presented as supplementary materials.

**Thank you for this suggestion. We have opted to keep Tables 1 and 2 as they are because we believe they demonstrate how the data are ‘high quality’ (see below our response to your question on that point) and provide information on how many outpatients attend the facilities each month. However, we have added unadjusted and adjusted IRRs to the following supplemental tables:**

**Study objective 1: S2 Table**

**Study objective 2: S3 Table**

• Please show the precision estimates for each point estimate of x% reduction or increase in IRRs.

**We have added 95% CI for the % reduction estimates in the results.**

• Please justify and discuss the choice of your analytical approach: mixed effects negative binomial regression models vs. interrupted time-series ( see example by Ashton et al 2019 below).

**We have opted to use this pre-post comparison using mixed effects models instead of interrupted time series methods due to the sample size. Since we only have 5 health facilities included in the analysis (meaning 5 observations per month), we would not have the power to accurately predict the counterfactual trend in the absence of IRS. Furthermore, given the temporal window of the analysis is quite long (7 years post-IRS implementation), and we had only 1 year of baseline data, we do not believe we have adequate baseline information to accurately predict counterfactual trends for a 7-year time period.**

• Please take this opportunity to highlight and strengthen the importance of using routine surveillance data in your discussion and citing relevant literature. Here are a few:

o Ashton RA. Use of routine health information system data to evaluate impact of malaria control interventions in Zanzibar, Tanzania from 2000 to 2015. EClinicalMedicine. 2019;12:11–9.
o Ashton R.A. et al. Methodological considerations for use of routine health information system data to evaluate malaria program impact in an era of declining malaria transmission. Am J Trop Med Hyg. 2017; 97: 46-57
o Bennett A. et al. A methodological framework for the improved use of routine health system data to evaluate national malaria control programs: evidence from Zambia. Popul Health Metr. 2014; 12: 30
o Bhattarai A, Ali AS, Kachur SP, Mårtensson A, Abbas AK, Khatib R, et al. (2007) Impact of Artemisinin-Based Combination Therapy and Insecticide-Treated Nets on Malaria Burden in Zanzibar. PLoS Med 4(11): e309.

**Thank you for pointing us to this literature. We have added the following citations to our manuscript:**

**Ashton RA, Bennett A, Al-Mafazy AW, Abass AK, Msellem MI, McElroy P, et al. Use of Routine Health Information System Data to Evaluate Impact of Malaria Control Interventions in Zanzibar, Tanzania from 2000 to 2015. EClinicalMedicine. 2019;12:11-9. Epub 2019/08/08. doi: 10.1016/j.eclinm.2019.05.011. PubMed PMID: 31388659; PubMed Central PMCID: PMC6677660.**

**Ashton RA, Bennett A, Yukich J, Bhattarai A, Keating J, Eisele TP. Methodological Considerations for Use of Routine Health Information System Data to Evaluate Malaria Program Impact in an Era of Declining Malaria Transmission. Am J Trop Med Hyg. 2017;97(3_Suppl):46-57. Epub 2017/10/11. doi: 10.4269/ajtmh.16-0734. PubMed PMID: 28990915; PubMed Central PMCID: PMC5619932.**

**Bennett A, Yukich J, Miller JM, Vounatsou P, Hamainza B, Ingwe MM, et al. A methodological framework for the improved use of routine health system data to evaluate national malaria control programs: evidence from Zambia. Popul Health Metr. 2014;12(1):30. Epub 2014/12/02. doi: 10.1186/s12963-014-0030-0. PubMed PMID: 25435815; PubMed Central PMCID: PMC4247605.**

**Alegana VA, Okiro EA, Snow RW. Routine data for malaria morbidity estimation in Africa: challenges and prospects. BMC Med. 2020;18(1):121. Epub 2020/06/04. doi: 10.1186/s12916-020-01593-y. PubMed PMID: 32487080; PubMed Central PMCID: PMC7268363.**

• Please also elaborate what “high quality data” means and how data quality of assessment was done for this analysis.

**The Introduction states:**

***UMSP places emphasis on high quality data, ensuring minimal missingness on key variables including age and place of residence, and providing training and materials to maximize diagnostic testing among patients suspected of having malaria.***

**Tables 1 and 2 give some indication of this; they demonstrate that testing rates for those suspected of having malaria are extremely high (>95% in most cases). We have also added the following sentence to the Discussion:**

***Unlike with standard HMIS data, UMSP surveillance sites have remarkably high reporting, testing, and proper treatment rates, with extremely low missingness of key variables; these data therefore lend themselves well to accurately assessing changes in malaria incidence over time.***

• The upper limit of 95% CI for non-IRS sites in year 6 is slightly different in the abstract (0.64) and results (0.63).

**Thank you for pointing out this error; it has been rectified.**
